# SRSF2 mutation reduces polycythemia and impairs hematopoietic progenitor functions in JAK2V617F-driven myeloproliferative neoplasm

**DOI:** 10.1038/s41408-023-00947-y

**Published:** 2023-11-27

**Authors:** Yue Yang, Salar Abbas, Mohammad A. Sayem, Avik Dutta, Golam Mohi

**Affiliations:** 1 Department of Biochemistry and Molecular Genetics, University of Virginia School of Medicine, Charlottesville, VA 22908 USA; 2 University of Virginia Cancer Center, Charlottesville, VA 22908 USA

**Keywords:** Myeloproliferative disease, Myeloproliferative disease

## Abstract

SRSF2 mutations are found in association with JAK2V617F in myeloproliferative neoplasms (MPN), most frequently in myelofibrosis (MF). However, the contribution of SRSF2 mutation in JAK2V617F-driven MPN remains elusive. To investigate the consequences of SRSF2^P95H^ and JAK2^V617F^ mutations in MPN, we generated Cre-inducible Srsf2^P95H/+^Jak2^V617F/+^ knock-in mice. We show that co-expression of Srsf2^P95H^ mutant reduced red blood cell, neutrophil, and platelet counts, attenuated splenomegaly but did not induce bone marrow fibrosis in Jak2^V617F/+^ mice. Furthermore, co-expression of Srsf2^P95H^ diminished the competitiveness of Jak2^V617F^ mutant hematopoietic stem/progenitor cells. We found that Srsf2^P95H^ mutant reduced the TGF-β levels but increased the expression of S100A8 and S100A9 in Jak2^V617F/+^ mice. Furthermore, enforced expression of S100A9 in Jak2^V617F/+^ mice bone marrow significantly reduced the red blood cell, hemoglobin, and hematocrit levels. Overall, these data suggest that concurrent expression of Srsf2^P95H^ and Jak2^V617F^ mutants reduces erythropoiesis but does not promote the development of bone marrow fibrosis in mice.

## Introduction

Myeloproliferative neoplasms (MPN) including polycythemia vera (PV), essential thrombocythemia (ET) and myelofibrosis (MF) are clonal myeloid malignancies derived from mutated hematopoietic stem cells [1]. The JAK2V617F is the most common somatic mutation associated with all three Philadelphia chromosome negative MPNs [1]. Although expression of heterozygous Jak2V617F mutant alone is sufficient to induce PV disease in mice [2–4], additional mutations or genetic events might be required for the development of MF. Interestingly, PV and ET can transform to MF following the acquisition of additional somatic mutations [5, 6].

Mutations in epigenetic modifiers (EZH2, ASXL1, DNMT3A and TET2) or spliceosome machinery (U2AF1 and SRSF2) were found in association with JAK2V617F in MF [5, 6]. Several studies using various mouse models have suggested that loss of Ezh2 (enhancer of zeste homologue 2), Asxl1 (additional sex combs-like 1) or Dnmt3a (DNA methyltransferase 3a) cooperates with Jak2V617F in the development of MF [7–11]. In contrast, concomitant Jak2V617F expression and Tet2 loss promotes MPN disease progression without manifesting bone marrow fibrosis in mice [12].

SRSF2 is a member of the serine/arginine-rich family of protein that is involved in RNA splicing [13]. SRSF2 mutations involving proline 95 residue (SRSF2P95) have been found in patients with various myeloid neoplasms including myelodysplastic syndromes (MDS), chronic myelomonocytic leukemia (CMML), MPN and acute myeloid leukemia (AML) [14, 15]. In MPN, SRSF2 mutations are rarely seen in PV and ET, but they occur in patients with MF and are associated with poor prognosis [5, 6]. Expression of Srsf2^P95H^ mutant in mice hematopoietic compartment results in myelodysplasia and impaired hematopoietic stem cell functions [16, 17]. However, the contribution of SRSF2 mutation in JAK2V617F-induced MPN remains unclear.

In this study, we investigated the effects of concurrent SRSF2^P95H^ and JAK2^V617F^ mutations in the pathogenesis of MPN using Cre-inducible Srsf2^P95H/+^Jak2^V617F/+^ knock-in mice. Results from our studies suggest that SRSF2 mutant inhibits erythropoiesis but does not promote the development of myelofibrosis in mice expressing Jak2V617F.

## Materials and methods

### Mice

Conditional Jak2^V617F^ knock-in [2], Srsf2^P95H^ knock-in [16] and Mx1Cre transgenic [18] mice were previously reported. All mice were on a C57BL/6 background. Mx1Cre expression was induced by intraperitoneal injection of three doses of 300 μg of polyinosine-polycytosine (pI-pC) at 4 weeks after birth. Wild type C57BL/6 and UBC-GFP mice were purchased from the Jackson Laboratory. All animal studies were performed in accordance with the guidelines approved by the Institutional Animal Care and Use Committee of University of Virginia School of Medicine.

### Bone marrow transplantation assays

For non-competitive BM transplantation (BMT) assays, BM (1 × 10^6^) cells from control, Srsf2^P95H/+^, Jak2^V617F/+^ or Srsf2^P95H/+^Jak2^V617F/+^ mice (without pI-pC) were transplanted into lethally irradiated C57BL/6 mice. Recipient animals were injected with three doses of 300 μg pI-pC at 4 weeks after transplantation. For competitive transplantation assays, BM cells from un-induced Jak2^V617F/+^ GFP+ or Srsf2^P95H/+^Jak2^V617F/+^ GFP+ mice were mixed with WT (non-GFP) competitor BM cells at 1:1 ratio and transplanted into lethally irradiated WT C57BL/6 recipient mice. At 4 weeks after transplantation, recipient animals were injected with three doses of 300 μg pI-pC. Chimerism was determined in the BM of transplanted animals by assessing the percentage of GFP+ cells.

### Blood and bone marrow analysis

Peripheral blood counts were measured using Hemavet 950FS (Drew Scientific). Mouse bone marrow specimens were fixed in 10% neutral buffered formalin and embedded in paraffin. Tissue sections (4μm) were stained with hematoxylin and eosin (H&E) and reticulin stains.

### Colony-forming assays

Mouse bone marrow (2 × 10^4^) cells were plated in complete methylcellulose medium (MethoCult M3434; StemCell Technologies) containing cytokines. Burst forming units-erythroid (BFU-E) and granulocyte-macrophage colony-forming units (CFU-GM) colonies were counted on day 7. To detect Epo-independent colony-forming units-erythroid (CFU-E) colonies, spleen cells (1 × 10^5^) were plated in MethoCult M3234 medium (StemCell Technologies) in the absence of cytokine. CFU-E colonies were counted after 2 days by staining with benzidine solution (Sigma-Aldrich). To determine colony-forming units-megakaryocytes (CFU-Mk), BM cells (1 × 10^5^) were plated in collagen-based MegaCult medium (StemCell Technologies) in the presence of Tpo, IL-3, IL-6 and IL-11. CFU-Mk colonies were scored after 8 days according to the protocol from StemCell Technologies. To evaluate the effect of S100A8 or S100A9 overexpression on CFU-GM and BFU-E formation of Jak2^V617F/+^ BM cells, lineage-negative cells were isolated from the BM of Jak2^V617F/+^ mice using a Lineage Cell Depletion Kit (#130-110-470, Miltenyi Biotec) and transduced with retroviruses expressing vector, S100A8 or S100A9 by two rounds of spin infection. Infected cells were selected using 2.5 μg/mL puromycin for 48 h and 2.5 × 10^3^ lineage-negative cells were plated in duplicates in cytokine-supplemented complete methylcellulose medium (MethoCult M3434; StemCell Technologies, Canada). CFU-GM and BFU-E colonies were scored on day 7.

### Plasmids

Mouse *S100A8* and *S100A9* cDNA constructs were purchased from OriGene and sub-cloned into pLZRS vector.

### Retroviral transduction and bone marrow transplantation

High-titer retroviral stocks of pLZRS-vector and pLZRS-mouse *S100A9* were prepared by transient transfection of Plat-E cells (CELL BIOLABS, CA). Bone marrow cells from 5-fluorouracil-treated Jak2^V617F/+^ mice were transduced with retroviruses expressing vector or S100A9 by two rounds of spin infection. One million transduced bone marrow cells were injected into retro-orbital veins of lethally irradiated (2 × 5.5 Gy) C57BL/6 recipient mice.

### Immunoblotting

BM cells were lysed in 2x sample buffer by direct boiling. Immunoblotting was performed using antibodies against S100A8 (AF3059, R&D, MN), S100A9 (AF2065, R&D, MN) and β-actin (A5441, Sigma, MO).

### Flow cytometry

For precursor cells analysis, bone marrow (BM) and spleen cells were stained for 30 min on ice with monoclonal antibodies against Ter119, CD71, CD41, Mac-1, Gr-1, CD45R (B220) or TCRβ. For hematopoietic stem/progenitor cell analysis, BM or spleen cells were stained for 60 min on ice with antibodies against lineage (Lin) markers (CD3e, CD4, CD8, CD19, B220, Gr-1, Ter119 and CD127), c-Kit, Sca-1, CD135, CD34 and CD16/32 (FcγRII/III). Flow cytometry antibodies were purchased from Invitrogen or BioLegend. Flow cytometry was performed with a BD Fortessa flow cytometer and analyzed using FlowJo 10 software (FlowJo, LLC).

### TGF-β1 Enzyme-linked immunosorbent assay (ELISA)

TGF-β1 levels in the serum of mice were determined using TGF-β1 ELISA kit (R&D Systems) according to the manufacturer’s protocols.

### Real-time quantitative PCR

Megakaryocytic-erythroid progenitors (MEP) were sorted from BM cells of control, Srsf2^P95H/+^, Jak2^V617F/+^ and Srsf2^P95H/+^Jak2^V617F/+^ mice using a FACS Aria II (BD, NJ). Total RNA was extracted from the MEPs with RNeasy micro kit (Qiagen, Germany) and cDNA samples were prepared by using QuantiTect Reverse Transcription kit (Qiagen, Germany). Real-time PCR was performed on a QuantStudio 3 system (Applied Biosystems, MA) machine using SYBR Green PCR master mix (Quantabio, MA). The data were normalized to *Hprt* and fold changes of mRNA expression were determined with the ΔΔCt method. Primers used for real-time PCR were: *S100a8*_Forward, 5′-ACAATGCCGTCTGAACTGG-3′; *S100a8*_Reverse, 5′-CTCTGCTACTCCTTGTGGCTGTC-3′; *S100a9*_Forward, 5′-CAGCATAACCACCATCATCG-3′; *S100a9*_Reverse, 5′-GTCCTGGTTTGTGTCCAGGT-3′; *Hprt*_Forward, 5′-CAACGGGGGACATAAAAGTTATTGGTGGA-3′; and *Hprt*_Reverse, 5′-TGCAACCTTAACCATTTTGGGGCTGT-3′.

### Statistical analysis

All statistical analyses were performed using the GraphPad Prism 9.4.1 (GraphPad Software). For comparisons between two groups, unpaired two-tailed Student’s *t* test was used. When comparing more than two groups, one-way ANOVA with Tukey’s multiple comparison test was used. All data are presented as mean ± SEM. *P* < 0.05 was considered statistically significant (*P < 0.05; **P < 0.01; ***P < 0.001; ****P < 0.0001).

## Results

### Srsf2^P95H^ mutant reduces polycythemia phenotype in Jak2^V617F^ knock-in mice

**Fig. 1:**
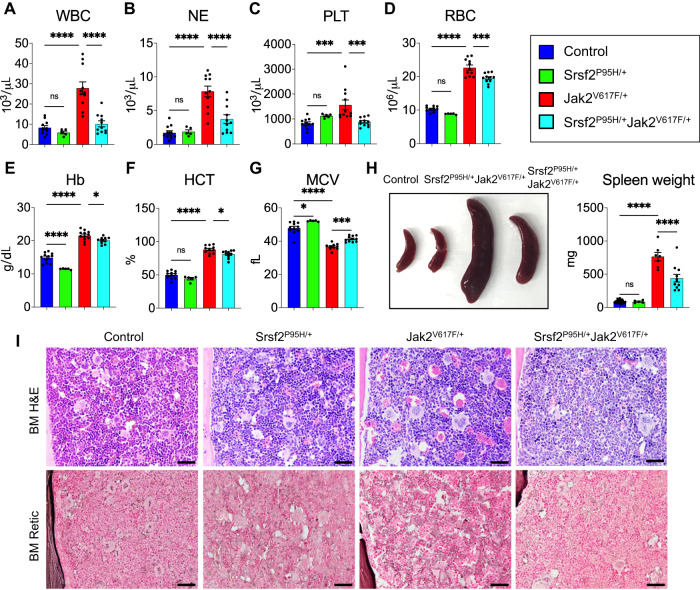
SRSF2^P95H^ mutant reduces polycythemia in Jak2^V617F^ knock-in mice. **A** White blood cell (WBC), **B** neutrophil (NE), **C** platelet (PLT), **D** red blood cell (RBC), **E** hemoglobin (Hb), **F** hematocrit (HCT) and **G** mean corpuscular volume (MCV) counts in the peripheral blood of control (*n* = 11), Srsf2^P95H/+^ (*n* = 5), Jak2^V617F/+^ (*n* = 11) and Srsf2^P95H/+^Jak2^V617F/+^ (*n* = 11) mice were assessed at 24 weeks after pI-pC induction. **H** Spleen size/weight in control (*n* = 11), Srsf2^P95H/+^ (*n* = 6), Jak2^V617F/+^ (*n* = 8) and Srsf2^P95H/+^Jak2^V617F/+^ (*n* = 11) mice. **I** Bone marrow histology. Representative images of the H&E and Reticulin staining of the BM sections from control, Srsf2^P95H/+^, Jak2^V617F/+^ and Srsf2^P95H/+^Jak2^V617F/+^ mice (*n* = 5–6 per group) at 24 weeks after pI-pC induction. Scale bar, 20 μm. Data are presented in bar graphs as mean ± SEM. (*P < 0.05; ***P < 0.001; ****P < 0.0001; ns not significant). Statistical significances were determined using one-way ANOVA with Tukey’s multiple comparison test.

In order to investigate the consequences of concurrent SRSF2^P95H^ and JAK2^V617F^ mutations in MPN, we generated Cre-inducible Srsf2^P95H/+^Jak2^V617F/+^ knock-in mice by crossing conditional Jak2^V617F^ knock-in mice [2] with conditional Srsf2^P95H^ knock-in [16] and Mx1Cre transgenic [18] mice. The expression of Srsf2^P95H/+^ and Jak2^V617F/+^ mutants were induced in the hematopoietic compartments of these mice at 4 weeks after birth following intraperitoneal injection of polyinosine-polycytosine (pI-pC). We analyzed four groups of mice: control (WT or Mx1Cre only), Mx1Cre; Srsf2^P95H/+^ (hereafter Srsf2^P95H/+^), Mx1Cre; Jak2^V617F/+^ (hereafter Jak2^V617F/+^) and Mx1Cre; Srsf2^P95H/+^Jak2^V617F/+^ (hereafter Srsf2^P95H/+^Jak2^V617F/+^) mice. Mice were analyzed at 24 weeks after pI-pC induction (i.e., at 28 weeks after birth). Consistent with our previous report [2], mice expressing heterozygous Jak2^V617F^ (Jak2^V617F/+^) showed a PV disease characterized by significant increase in white blood cell (WBC), neutrophil (NE), platelet (PLT), red blood cell (RBC), hemoglobin (Hb) and hematocrit (HCT) levels in their peripheral blood compared to control mice (Fig. 1A–F). Mice expressing heterozygous Srsf2^P95H^ (Srsf2^P95H/+^) displayed decreased hemoglobin but increased mean corpuscular volume (MCV) relative to control mice (Fig. 1E, G), consistent with published report [16]. Srsf2^P95H/+^Jak2^V617F/+^ mice expressing both Srsf2^P95H^ and Jak2^V617F^ mutants exhibited significantly reduced WBC, neutrophil, platelet, RBC, hemoglobin and hematocrit parameters compared to Jak2^V617F/+^ mice (Fig. 1A–F). While Jak2^V617F/+^ mice displayed significantly reduced MCV, Srsf2^P95H/+^Jak2^V617F/+^ mice had higher MCV values compared to Jak2^V617F/+^ mice (Fig. 1G). Jak2^V617F/+^ mice exhibited marked splenomegaly, whereas Srsf2^P95H/+^Jak2^V617F/+^ mice had significantly reduced spleen size/weight compared to Jak2^V617F/+^ mice (Fig. 1H). H&E staining of the BM sections from WT (control) and Srsf2^P95H/+^ mutant mice showed normal BM cellularity (Fig. 1I). Jak2^V617F/+^ mice BM sections exhibited hypercellularity with significant increase in erythroid precursors and megakaryocyte clusters (Fig. 1I). Srsf2^P95H/+^Jak2^V617F/+^ mice BM sections exhibited normal BM cellularity and a reduction of erythroid precursors and megakaryocyte clusters compared to Jak2^V617F/+^ mice BM (Fig. 1I). Reticulin staining of the BM sections from Jak2^V617F/+^ mice showed mild to no bone marrow fibrosis at 24 weeks after induction (Fig. 1I). Srsf2^P95H/+^Jak2^V617F/+^ mice also did not exhibit bone marrow fibrosis at this time (Fig. 1I). A few Srsf2^P95H/+^Jak2^V617F/+^ mice were monitored for longer period and they were assessed for bone fibrosis at one year after induction. We also did not observe bone marrow fibrosis in Srsf2^P95H/+^Jak2^V617F/+^ mice at one year after induction (data not shown). Together, these results suggest that co-expression of Srsf2^P95H^ reduces polycythemia but does not promote myelofibrosis in Jak2^V617F/+^ mice.

### Effects of concurrent Srsf2^P95H^ and Jak2^V617F^ mutations on hematopoietic stem/progenitors and precursor cells in mice

**Fig. 2:**
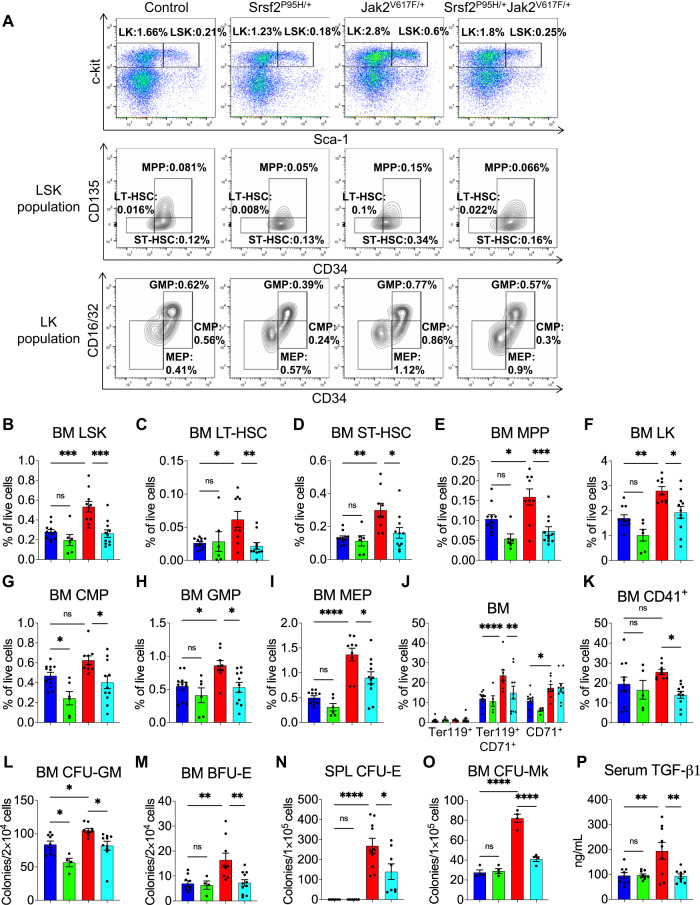
Effects of concurrent SRSF2^P95H^ and Jak2^V617F^ mutations on hematopoietic stem/progenitors and precursor cells. **A** Representative plots of flow cytometric analysis of control, Srsf2^P95H/+^, Jak2^V617F/+^ and Srsf2^P95H/+^Jak2^V617F/+^ mice are shown. Percentages of **B** LSK (Lin^−^Sca-1^+^c-kit^+^), **C** LT-HSC (Lin^-^Sca-1^+^c-kit^+^CD34^−^CD135^−^), **D** ST-HSC (Lin^−^Sca-1^+^c-kit^+^CD34^+^CD135^−^), **E** MPP (Lin^-^Sca-1^+^c-kit^+^CD34^+^CD135^+^), **F** LK (Lin^-^Sca-1^-^c-kit^+^), **G** CMP (Lin^-^Sca-1^-^c- kit^+^CD34^+^CD16/32^Low^), **H** GMP (Lin^-^Sca-1^-^c-kit^+^CD34^+^CD16/32^High^) and **I** MEP (Lin^−^Sca-1^−^c-kit^+^CD34^−^CD16/32^−^) in the BM of control (*n* = 11), Srsf2^P95H/+^ (*n* = 6), Jak2^V617F/+^ (*n* = 10) and Srsf2^P95H/+^Jak2^V617F/+^ (*n* = 11) mice are shown in bar graphs as mean ± SEM. **J** Flow cytometric analysis of erythroid precursors using surface marker Ter119 and CD71 in the BM of control (*n* = 11), Srsf2^P95H/+^ (*n* = 6), Jak2^V617F/+^ (*n* = 10) and Srsf2^P95H/+^Jak2^V617F/+^ (*n* = 11) mice are shown in bar graphs as mean ± SEM. **K** Percentages of CD41^+^ megakaryocytic precursors in the BM of control (*n* = 11), Srsf2^P95H/+^ (*n* = 5), Jak2^V617F/+^ (*n* = 9) and Srsf2^P95H/+^Jak2^V617F/+^ (*n* = 10) mice are shown in bar graphs as mean ± SEM. **L**, **M** Hematopoietic progenitor colony assays. In total, 2 × 10^4^ BM cells from control (*n* = 9), Srsf2^P95H/+^ (*n* = 4), Jak2^V617F/+^ (*n* = 9) and Srsf2^P95H/+^Jak2^V617F/+^ (*n* = 10) mice were plated in methylcellulose medium supplemented with cytokines. CFU-GM (**L**) and BFU-E (**M**) colonies were scored 7 days after plating. **N** Erythropoietin-independent CFU-E colony formation assay. In total, 1 × 10^5^ spleen cells from control (*n* = 6), Srsf2^P95H/+^ (*n* = 6), Jak2^V617F/+^ (*n* = 10) and Srsf2^P95H/+^Jak2^V617F/+^ (*n* = 8) mice were plated in methylcellulose medium without any cytokines. CFU-E colonies were scored after 2 days. **O** CFU-Mk colonies derived from the BM of control (*n* = 4), Srsf2^P95H/+^ (*n* = 4), Jak2^V617F/+^ (*n* = 4) and Srsf2^P95H/+^Jak2^V617F/+^ (*n* = 4) mice. **P** Serum TGF-β1 levels in control (*n* = 9), Srsf2^P95H/+^ (*n* = 9), Jak2^V617F/+^ (*n* = 9) and Srsf2^P95H/+^Jak2^V617F/+^ (*n* = 9) mice were assessed by ELISA. (*P < 0.05; **P < 0.01; ***P < 0.001; ****P < 0.0001; ns not significant). Statistical significances were determined using one-way ANOVA with Tukey’s multiple comparison test.

We next assessed the effects of concurrent expression of Srsf2^P95H^ and Jak2^V617F^ mutants on mice hematopoietic stem/progenitor cells (HSPC) by flow cytometry. The representative flow cytometry plots of HSPC analysis are depicted in Fig. 2A. Jak2^V617F/+^ mice exhibited significant increase in frequencies and total numbers of LSK (Lin^-^Sca-1^+^c-kit^+^), LT-HSC (long-term hematopoietic stem cells), ST-HSC (short-term hematopoietic stem cells) and MPP (multipotent progenitors) in their BM, while concurrent expression of Srsf2^P95H^ and Jak2^V617F^ significantly reduced the frequencies and total numbers of LSK, LT-HSC, ST-HSC and MPP populations in the BM of Srsf2^P95H/+^Jak2^V617F/+^ mice (Fig. 2B–E and Supplementary Fig. 1A–D). Co-expression of Srsf2^P95H^ and Jak2^V617F^ mutants also significantly reduced the frequencies and total numbers of LK (Lin^-^c-kit^+^; myeloid progenitors), CMP (common myeloid progenitors), GMP (granulocyte-macrophage progenitors) and MEP (megakaryocyte-erythroid progenitors) in the BM of Srsf2^P95H/+^Jak2^V617F/+^ mice compared to Jak2^V617F/+^ mice (Fig. 2F–I and Supplementary Fig. 1E–H). However, we did not observe significant changes of HSPC in the spleens of Srsf2^P95H/+^Jak2^V617F/+^ mice compared to Jak2^V617F/+^ mice (Supplementary Fig. 2A–H).

While Jak2^V617F/+^ mice BM exhibited a significant increase of erythroid precursors (Ter119^+^/CD71^+^) compared to control mice, Srsf2^P95H/+^Jak2^V617F/+^ mice BM showed significantly reduced erythroid precursors (Ter119^+^/CD71^+^) compared to Jak2^V617F/+^ mice (Fig. 2J). The Srsf2^P95H/+^Jak2^V617F/+^ mice also had reduced percentage of megakaryocytic (CD41^+^) cells in their BM compared to Jak2^V617F/+^ mice (Fig. 2K). Hematopoietic progenitor colony assays showed significantly increased number of CFU-GM and BFU-E colonies derived from the BM of Jak2^V617F/+^ mice compared to control animals, while the number of CFU-GM and BFU-E colonies derived from the BM of Srsf2^P95H/+^Jak2^V617F/+^ mice were significantly lower compared to Jak2^V617F/+^ mice (Fig. 2L, M). Spleens from Jak2^V617F/+^ mice exhibited large numbers of Epo-independent CFU-E colonies (Fig. 2N), a hallmark feature of PV [19], whereas spleens from Srsf2^P95H/+^Jak2^V617F/+^ mice had significantly reduced Epo-independent CFU-E colonies compared to Jak2^V617F/+^ mice (Fig. 2N). The number of CFU-Mk colonies derived from the BM of Jak2^V617/+^ mice was significantly higher compared to control animals (Fig. 2O). Srsf2^P95H/+^Jak2^V617F/+^ mice BM exhibited significantly reduced number of CFU-Mk colonies compared to Jak2^V617F/+^ mice (Fig. 2O). Aberrant expression of transforming growth factor beta 1 (TGF-β1) has been linked to MF [1, 20]. So, we assessed the TGF-β1 levels by ELISA. Whereas Jak2^V617F/+^ mice exhibited elevated levels of serum TGF-β1, Srsf2^P95H/+^Jak2^V617F/+^ mice showed significantly reduced serum TGF-β1 levels compared to Jak2^V617F/+^ mice (Fig. 2P).

### Phenotypes observed in Srsf2^P95H/+^Jak2^V617F/+^ mice are cell autonomous

**Fig. 3:**
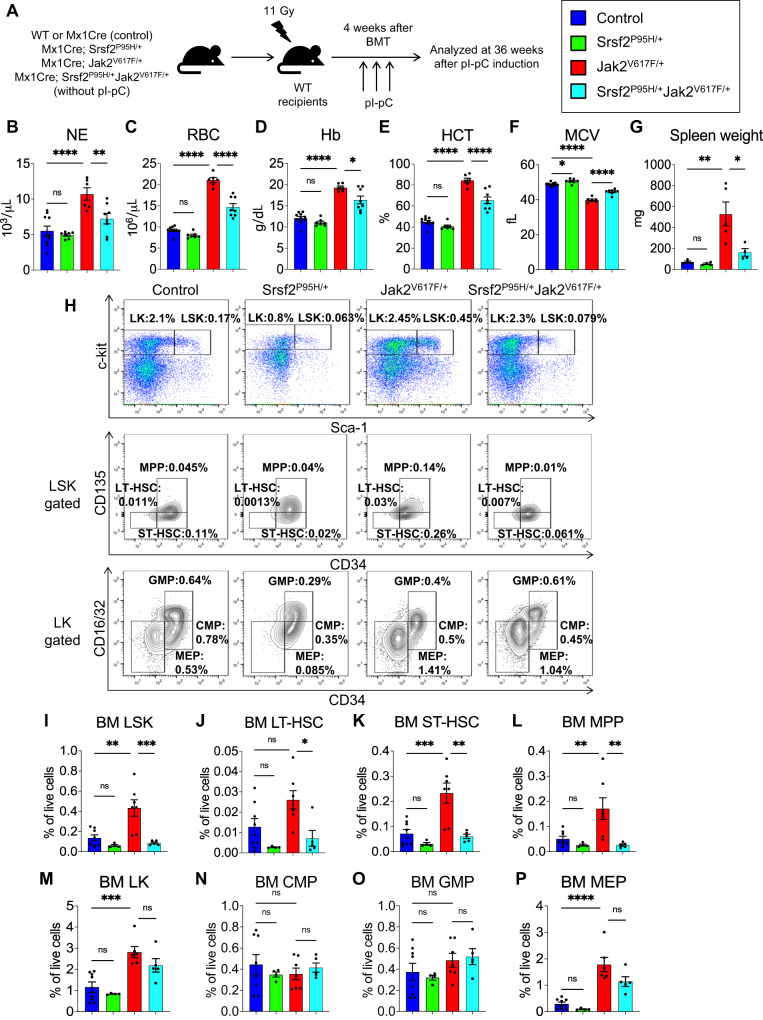
Phenotypes observed in the SRSF2^P95H/+^Jak2^V617F/+^ mice are cell autonomous. **A** Experimental design for cell autonomous bone marrow transplantation (BMT) assay. BM cells from control, Srsf2^P95H/+^, Jak2^V617F/+^ and Srsf2^P95H/+^Jak2^V617F/+^ mice at 8 weeks after birth (without pI-pC) were transplanted into lethally irradiated wild type C57BL/6 recipient mice (1 × 10^6^ cells/recipient). At 4 weeks after BMT, pI-pC injections were given to induce the expression of Srsf2^P95H^ and Jak2^V617F^ mutants in the recipient animals. Recipient mice were analyzed at 36 weeks after pI-pC induction. Peripheral blood **B** neutrophil (NE), **C** red blood cell (RBC), **D** hemoglobin (Hb), **E** hematocrit (HCT) and **F** mean corpuscular volume (MCV) counts of control (*n* = 10), Srsf2^P95H/+^ (*n* = 7), Jak2^V617F+^ (*n* = 6) and Srsf2^P95H/+^Jak2^V617F/+^ (*n* = 8) mice are shown in bar graphs. **G** Spleen weights of control (*n* = 4), Srsf2^P95H/+^ (*n* = 4), Jak2^V617F/+^ (*n* = 5) and Srsf2^P95H/+^Jak2^V617F/+^ mice (*n* = 4). **H** Representative plots of flow cytometric analysis of control, Srsf2^P95H/+^, Jak2^V617F/+^ and Srsf2^P95H/+^Jak2^V617F/+^ BMT mice. Frequencies of **I** LSK, **J** LT-HSC, **K** ST-HSC, **L** MPP, **M** LK, **N** CMP, **O** GMP and **P** MEP in the BM of control (*n* = 8), Srsf2^P95H/+^ (*n* = 4), Jak2^V617F/+^ (*n* = 7) and Srsf2^P95H/+^Jak2^V617F/+^ mice (*n* = 5) are shown in bar graphs as mean ± SEM. (*P < 0.05; **P < 0.01; ***P < 0.001; ****P < 0.0001; ns not significant). Statistical significances were determined using one-way ANOVA with Tukey’s multiple comparison test.

To assess whether the phenotypes observed in Srsf2^P95H/+^Jak2^V617F/+^ mice were cell intrinsic, we transplanted BM cells from control, Srsf2^P95H/+^, Jak2^V617F/+^ and Srsf2^P95H/+^Jak2^V617F/+^ mice into lethally irradiated C57BL/6 wild type recipient mice as outlined in Fig. 3A. At 4 weeks after transplantation, mice were injected with pI-pC to induce expression of Srsf2^P95H/+^ and Jak2^V617F/+^ in hematopoietic compartments. Transplanted animals expressing Jak2^V617F/+^ exhibited elevated neutrophil (NE), red blood cell (RBC), hemoglobin (Hb) and hematocrit (HCT) levels but decreased MCV in the peripheral blood compared to control animals (Fig. 3B–F). Co-expression of Srsf2^P95H/+^ and Jak2^V617F/+^ mutants significantly reduced neutrophil, RBC, hemoglobin and hematocrit levels but increased MCV in the recipient animals compared to mice expressing Jak2^V617F/+^ (Fig. 3B–F). Transplanted animals expressing Jak2^V617F/+^ showed marked splenomegaly, whereas mice co-expressing Srsf2^P95H/+^ and Jak2^V617F/+^ mutants exhibited significantly reduced spleen weights compared to Jak2^V617F/+^ mice (Fig. 3G). Flow cytometry analyses showed decreased percentages of LSK, LT-HSC, ST-HSC and MPP in the BM of transplanted mice co-expressing Srsf2^P95H/+^ and Jak2^V617F/+^ compared to mice expressing Jak2^V617F/+^ (Fig. 3H–L). The percentages of LSK, LT-HSC and MPP were also significantly reduced in the spleens of transplanted mice co-expressing Srsf2^P95H/+^ and Jak2^V617F/+^ compared to mice expressing Jak2^V617F/+^ (Supplementary Fig. 3A–D). However, the percentages of LK, CMP, GMP and MEP were not significantly altered in the BM of transplanted mice co-expressing Srsf2^P95H/+^ and Jak2^V617F/+^ compared to mice expressing Jak2^V617F/+^ (Fig. 3M–P).

### Expression of Srsf2^P95H^ mutant reduces the competitiveness of Jak2^V617F^ HSPC

**Fig. 4:**
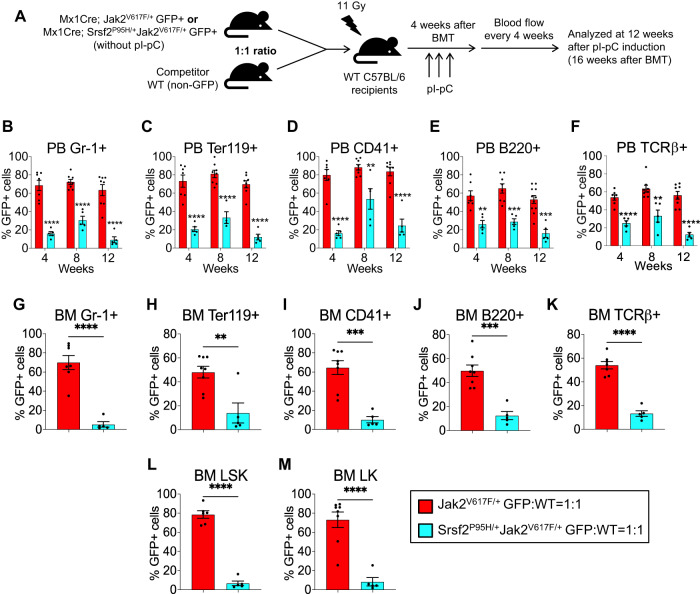
Srsf2^P95H^ mutant reduces the competitiveness of Jak2^V617F^ HSPC. **A** A scheme on competitive BM transplantation assay. BM cells (5 × 10^5^) from Jak2^V617F/+^GFP^+^ or Srsf2^P95H/+^Jak2^V617F/+^GFP^+^ mice without pI-pC injection were mixed with non-GFP WT BM (5 × 10^5^) cells at a 1:1 ratio and transplanted into lethally irradiated non-GFP WT C57BL/6 recipient mice. pI-pC injections were given to the recipients at 4 weeks after BMT to induce Srsf2^P95H^ and Jak2^V617F^ expression. The recipient mice were analyzed at 12 weeks after pI-pC injections. Percentages of donor derived (GFP + ) **B** Gr-1^+^, **C** Ter119^+^, **D** CD41^+^, **E** B220^+^ and **F** TCRβ^+^ cells in the peripheral blood of recipients at 4, 8 and 12 weeks after pI-pC injections are shown in bar graphs as mean ± SEM. Percentages of GFP+ **G** Gr-1^+^, **H** Ter119^+^, **I** CD41^+^, **J** B220^+^, **K** TCRβ^+^, **L** LSK and **M** LK cells in the BM of recipient mice are shown in bar graphs as mean ± SEM (Jak2^V617F/+^GFP: WT = 1:1, *n* = 6–8; Srsf2^P95H/+^Jak2^V617F/+^GFP: WT = 1:1, *n* = 5). (*P < 0.05; **P < 0.01; ***P < 0.001; ****P < 0.0001). Statistical significances were determined using two-tailed unpaired *t* test.

To evaluate the effects of concurrent expression of Srsf2^P95H^ and Jak2^V617F^ mutants on HSPC function, we performed competitive repopulation assays (outlined in Fig. 4A). We generated Mx1Cre; Jak2^V617F/+^ GFP+ and Mx1Cre; Srsf2^P95H/+^Jak2^V617F/+^ GFP+ mice. Equal numbers of BM cells from these donor mice (5 × 10^5^) were mixed with WT (non-GFP) mice BM cells (5 × 10^5^) at a ratio of 1:1 and then transplanted into lethally irradiated WT C57BL/6 mice. At 4 weeks after BMT, the recipient animals were injected with pI-pC to induce Srsf2^P95H/+^ and Jak2^V617F/+^ expression. The percentages of donor-derived mutant (GFP + ) cells were determined in the peripheral blood leukocytes of the chimeric mice by flow cytometry every 4 weeks and the mice were analyzed at 12 weeks after pI-pC induction (i.e., 16 weeks after BMT). We observed significantly higher percentages of GFP+ granulocyte (Gr-1^+^), erythroid (Ter119^+^), megakaryocyte (CD41^+^), B-lymphocyte (B220^+^) and T-lymphocyte (TCRβ^+^) cells in the peripheral blood of chimeric mice receiving Jak2^V617F/+^ BM compared with chimeric mice receiving Srsf2^P95H/+^Jak2^V617F/+^ BM (Fig. 4B–F). We also observed significantly reduced percentages of GFP+ Gr-1^+^, Ter119^+^, CD41^+^, B220^+^ and TCRβ^+^ cells in the BM of chimeric recipient animals receiving Srsf2^P95H/+^Jak2^V617F/+^ BM compared with Jak2^V617F/+^ BM (Fig. 4G–K). Similarly, we observed significantly reduced percentages of GFP+ Gr-1^+^, Ter119^+^, CD41^+^, B220^+^ and TCRβ^+^ cells in the spleens of chimeric animals receiving Srsf2^P95H/+^Jak2^V617F/+^ BM compared with Jak2^V617F/+^ BM (Supplementary Fig. 4A–E). Whereas the majority (70–80%) of LSK and LK cells in the BM and spleens of chimeric animals receiving Jak2^V617F/+^ BM were GFP+ at 12 weeks after pI-pC induction (16 weeks after transplantation), the percentages of GFP + LSK and LK cells in the BM and spleens were significantly lower in chimeric animals receiving Srsf2^P95H/+^Jak2^V617F/+^ BM compared with Jak2^V617F/+^ BM (Fig. 4L, M and Supplementary Fig. 4F, G). These data suggest that co-expression of Srsf2^P95H^ mutant reduces the hematopoietic progenitor function and diminishes the clonal advantage of Jak2^V617F^ mutant HSPC.

### Srsf2^P95H^ mutant-induced overexpression of S100A9 contributes to decreased erythropoiesis in Jak2^V617F/+^ mice

**Fig. 5:**
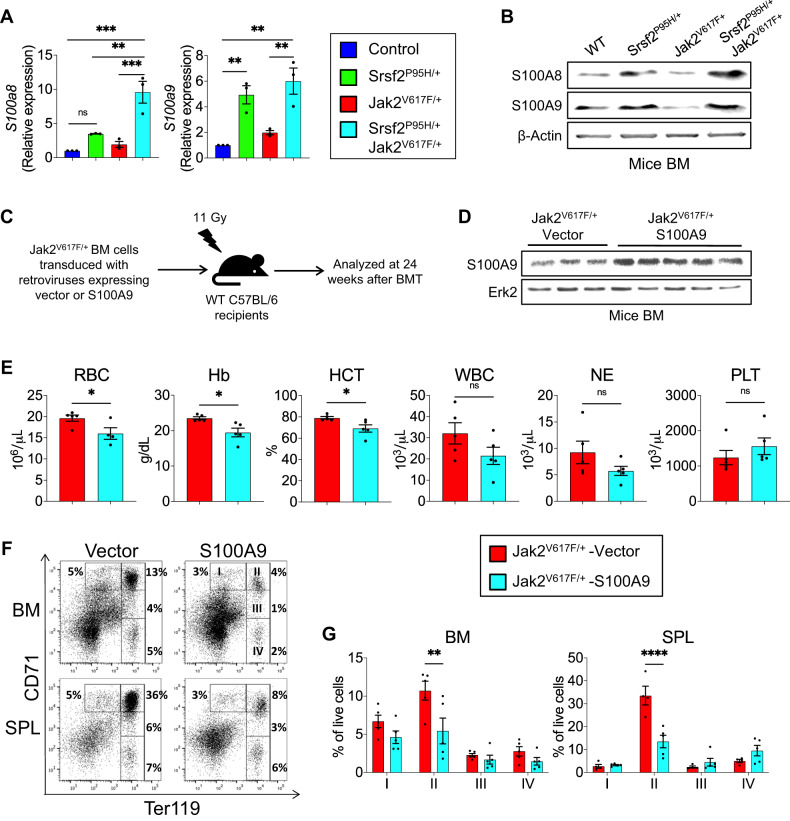
S100A9 overexpression induced by Srsf2^P95H^ contributes to decreased erythrocytosis in Jak2^V617F/+^ mice. **A** mRNA expression of *S100a8* and *S100a9* was determined in sorted MEP from the BM of control, Srsf2^P95H/+^, Jak2^V617F/+^ and Srsf2^P95H/+^Jak2^V617F/+^ mice by RT-qPCR. Relative expression levels were normalized to housekeeping gene *Hprt* (*n* = 3). **B** Immunoblots showing increased expression of S100A8 and S100A9 proteins in Srsf2^P95H/+^Jak2^V617F/+^ BM compared with WT (control) and Jak2^V617F/+^ BM. β-actin was used as a loading control. **C** A scheme on the experimental design is depicted. Jak2^V617F/+^ BM cells were transduced with retroviruses expressing vector or S100A9 and transplanted into lethally irradiated C57BL/6 mice. The recipient mice were analyzed at 24 weeks after BMT. **D** Immunoblot analysis of S100A9 expression in the Jak2^V617F/+^ BM transplanted animals expressing vector or S100A9. Erk2 was used as a loading control. **E** Peripheral blood RBC, Hb, HCT, WBC, NE and PLT counts of transplanted mice receiving Jak2^V617F/+^ BM expressing vector (*n* = 5) or S100A9 (*n* = 5) were measured at 24 weeks after BMT. **F** Representative plots of flow cytometric analysis of erythroid precursors in the BM and spleens of recipient mice expressing vector or S100A9 using surface markers CD71 and Ter119. **G** Percentages of erythroid precursor cells at different stages of differentiation (stage I–IV, from immature to mature) are shown in bar graphs as mean ± SEM. (*P < 0.05; **P < 0.01; ***P < 0.001; ****P < 0.0001; ns not significant). Significance was determined in (**A**) using one-way ANOVA with Tukey’s multiple comparison test. Statistical significances in (**E**, **G**) were determined using two-tailed unpaired *t* test.

A previous study has shown increased expression of *S100a8* and *S100a9* mRNA in hematopoietic progenitors of Srsf2^P95H/+^ mice [21]. Furthermore, increased expression of S100A8 and S100A9 has been linked to erythroid differentiation defects and MDS pathogenesis [22, 23]. Since we observed decreased erythrocytosis in Srsf2^P95H/+^Jak2^V617F/+^ mice, we assessed the expression of S100A8 and S100A9 in MEP (megakaryocyte-erythroid progenitors) by RT-qPCR. We found significantly increased expression of *S100a8* and *S100a9* mRNA in Srsf2^P95H/+^Jak2^V617F/+^ mice MEP compared with Jak2^V617F/+^ mice MEP (Fig. 5A). We also observed significantly increased expression of *S100a9* mRNA in Srsf2^P95H/+^ mice MEP compared with WT mice MEP (Fig. 5A). Immunoblot analyses also revealed increased expression of S100A8 and S100A9 proteins in the BM of Srsf2^P95H/+^ and Srsf2^P95H/+^Jak2^V617F/+^ mice compared with WT or Jak2^V617F/+^ mice BM (Fig. 5B). We further performed functional validation by retroviral overexpression of S100A8 and S100A9 into Jak2^V617F/+^ mice BM and progenitor colony assays. We observed significantly reduced CFU-GM and BFU-E colony formation by overexpression of S100A8 and S100A9 in the BM of Jak2^V617F/+^ mice (Supplementary Fig. 5A, B).

To assess the in vivo effects of S100A9 overexpression in Jak2^V617F/+^ mice, we performed bone marrow transplantation assays following retroviral expression of empty vector or S100A9 into Jak2^V617F/+^ BM cells (outlined in Fig. 5C). Immunoblot analysis confirmed increased S100A9 protein levels in the BM of Jak2^V617F/+^ mice expressing S100A9 (Fig. 5D). Transplanted animals receiving Jak2^V617F/+^ BM overexpressing S100A9 exhibited significantly reduced RBC, hemoglobin and hematocrit counts compared to recipients of Jak2^V617F/+^ BM expressing vector at 24 weeks after transplantation (Fig. 5E). However, WBC, neutrophil and platelet counts were not significantly altered by S100A9 overexpression (Fig. 5E). Flow cytometric analysis showed that recipients of Jak2^V617F/+^ BM expressing S100A9 had reduced erythroid precursors (stage II, CD71^high^Ter119^high^) in their BM and spleens compared to recipient animals expressing vector (Fig. 5F, G). However, we did not observe bone marrow fibrosis in transplanted animals receiving S100A9 transduced Jak2^V617F/+^ mice BM (data not shown). Together, these results suggest that Srsf2^P95H^ mutant induces overexpression of S100A9 (and S100A8) and contributes to reduced erythropoiesis in Jak2^V617F/+^ mice.

## Discussion

SRSF2 mutations have been found in association with JAK2V617F in patient with MF and linked to poor survival [5, 6]. However, the contribution of these two co-occurring mutations in MF has remained elusive. In this report, we show that concurrent expression of Srsf2^P95H^ mutant reduces peripheral blood neutrophil, RBC, hemoglobin, hematocrit and platelet counts and attenuates extramedullary hematopoiesis in Jak2^V617F/+^ mice. Notably, mice co-expressing Srsf2^P95H^ and Jak2^V617F^ mutants did not develop bone marrow fibrosis. These results are in contrast to other studies indicating cooperative effects of loss of Ezh2, Asxl1 or Dnmt3a with Jak2^V617F^ in the development of myelofibrosis in mice [7–11].

Several Srsf2^P95H/+^ knock-in mouse models have been reported [16, 17, 24]. Expression of Srsf2^P95H/+^ in mice hematopoietic compartment results in leukopenia, anemia and impaired hematopoietic stem cell self-renewal [16, 17]. Consistent with this, we have observed significant decrease of LSK, LT-HSC, ST-HSC and MPP in the BM and spleens of transplanted animals expressing Srsf2^P95H/+^Jak2^V617F/+^ compared to Jak2^V617F/+^ (Fig. 3I–L and Supplementary Fig. 3A–D). We also observed significant reduction of HSPCs in the BM of primary Srsf2^P95H/+^Jak2^V617F/+^ mice compared to Jak2^V617F/+^ mice. There was a trend of decreased LSK, LT-HSC, ST-HSC and MPP in the spleens of primary Srsf2^P95H/+^Jak2^V617F/+^ mice compared to Jak2^V617F/+^ mice although it did not reach to significance, indicating that more time might be required for significant reduction of HSPCs in the spleens of primary Srsf2^P95H/+^Jak2^V617F/+^ mice. It also has been reported that Srsf2^P95H/+^ promotes myeloid biased hematopoiesis [24]. If the competitor cells are from another age-matched control mice, Srsf2^P95H/+^ HSCs show significantly impaired competitive repopulation ability. If the competitor cells are matched for age and microenvironment, Srsf2^P95H/+^ cells can outcompete WT cells [24]. Previous studies have suggested that Jak2^V617F^ mutant confers clonal advantage to HSPC [7, 25, 26]. In the present study, we observed that Srsf2^P95H^ mutant reduced the competitiveness of Jak2^V617F^ mutant HSPC. We also did not see progression to myelofibrosis in mice co-expressing Srsf2^P95H^ and Jak2^V617F^ mutants. Thus, SRSF2^P95H^ mutant may contribute to ineffective hematopoiesis rather than bone marrow fibrosis in JAK2V617F-positive MPN.

TGF-β signaling has been linked to various tissue fibrosis [27, 28]. Increased levels of TGF-β1 have been observed in patients with MF as well as in mouse models of MF [29–32]. We observed significantly reduced serum TGF-β1 levels in Srsf2^P95H/+^Jak2^V617F/+^ mice compared to Jak2^V617F/+^ mice. This may explain the lack of bone marrow fibrosis in Srsf2^P95H/+^Jak2^V617F/+^ mice. Interestingly, we observed increased expression of S100A8 and S100A9 in Srsf2^P95H/+^Jak2^V617F/+^ mice BM compared to Jak2^V617F/+^ mice BM. A previous study reported increased expression of *S100a8* and *S100a9* mRNA in hematopoietic progenitors of Srsf2^P95H/+^ mice [21]. It has been suggested that increased expression of S100A8 and S100A9 contributes to erythroid differentiation defects and MDS pathogenesis [22, 23]. Transgenic mice expressing S100A9 exhibit ineffective hematopoiesis and MDS-like phenotype [33]. We found that retroviral overexpression of S100A9 into Jak2^V617F/+^ BM cells results in significantly lower blood RBC, hemoglobin and hematocrit counts, reduced erythroid precursors in the BM and decreased erythroid (BFU-E) colony formation. However, overexpression of S100A9 in Jak2^V617F/+^ mice BM did not induce myelofibrosis in transplanted animals (data not shown). Thus, increased expression of S100A9 (and S100A8) induced by SRSF2^P95H^ mutant may contribute to impaired erythropoiesis in JAK2V617F-positive MPN.

In conclusion, we demonstrate that Srsf2^P95H^ mutant reduces polycythemia and impairs competitiveness of Jak2^V617F^ mutant hematopoietic stem/progenitor cells but does not promote the development of bone marrow fibrosis in Jak2^V617F^-induced MPN. Similar observations have been made in a recent study by Willekens et al. [34]. Additional mutations or genetic abnormalities are required in association with SRSF2^P95H^ and JAK2^V617F^ mutations in the development of full-blown myelofibrosis.

### Supplementary information

Supplementary Information

## Data Availability

The datasets generated during the current study are available from the corresponding author on reasonable request.
