# Supplementary material for: SRSF2 mutation reduces polycythemia and impairs hematopoietic progenitor functions in JAK2V617F-driven myeloproliferative neoplasm
Source: Blood Cancer J. 2023 Nov 27;13(1):171. doi: 10.1038/s41408-023-00947-y (PMC10682023; doi:10.1038/s41408-023-00947-y)

Supplementary Information

Yang et. al.

SUPPLEMENTARY FIGURE LEGEND

Supplementary Figure 1. Effects of concurrent expression Srsf2^{P95H} and Jak2^{V617F} mutants on BM hematopoietic stem/progenitor cells

Total numbers of (A) LSK, (B) LT-HSC, (C) ST-HSC, (D) MPP, (E) LK, (F) CMP, (G) GMP and (H) MEP in the bone marrow of control (n=11), Srsf2$^{P95H/+}$ (n=6), Jak2$^{V617F/+}$ (n=10) and Srsf2$^{P95H/+}$Jak2$^{V617F/+}$ (n=11) mice are shown in bar graphs as mean ± SEM. (* p<0.05; ** p<0.01; *** p<0.001; **** p<0.0001). Significances were determined using one-way ANOVA with Tukey's multiple comparisons test.

Supplementary Figure 2. Effects of concurrent expression Srsf2^{P95H} and Jak2^{V617F} mutants on hematopoietic stem/progenitor cells in the spleens of primary mice

Percentages of (A) LSK (Lin$^-$Sca-1$^+$c-kit$^+$), (B) LT-HSC (Lin$^-$Sca-1$^+$c-kit$^+$CD34$^-$CD135$^-$), (C) ST-HSC (Lin$^-$Sca-1$^+$c-kit$^+$CD34$^+$CD135$^-$), (D) MPP (Lin$^-$Sca-1$^+$c-kit$^+$CD34$^+$CD135$^+$), (E) LK (Lin$^-$Sca-1$^-$c-kit$^+$), (F) CMP (Lin$^-$Sca-1$^-$c-kit$^+$CD34$^+$CD16/32^{low}), (G) GMP (Lin$^-$Sca-1$^-$c-kit$^+$CD34$^+$CD16/32^{High}) and (H) MEP (Lin$^-$Sca-1$^-$c-kit$^+$CD34$^-$CD16/32$^-$) in the spleens of control (n=11), Srsf2$^{P95H/+}$ (n=5), Jak2$^{V617F/+}$ (n=9) and Srsf2$^{P95H/+}$Jak2$^{V617F/+}$ (n=11) mice are shown in bar graphs as mean ± SEM. (* p<0.05; ** p<0.01; **** p<0.0001; ns, not significant). Significances were determined using one-way ANOVA with Tukey's multiple comparisons test.

Supplementary Figure 3. Effects of concurrent expression Srsf2^{P95H} and Jak2^{V617F} on hematopoietic stem/progenitor cells in the spleens of transplanted mice

Percentages of (A) LSK, (B) LT-HSC, (C) ST-HSC and (D) MPP in the spleens of control (n=8), Srsf2$^{P95H/+}$ (n=4), Jak2$^{V617F/+}$ (n=4) and Srsf2$^{P95H/+}$Jak2$^{V617F/+}$ (n=5) BMT mice are shown in bar graphs as mean ± SEM. (* p<0.05; ** p<0.01; *** p<0.001; **** p<0.0001; ns, not significant). Significances were determined using one-way ANOVA with Tukey's multiple comparisons test.

Supplementary Figure 4. Effects of Srsf2^{P95H} mutation on the repopulation capacity of Jak2$^{V617F/+}$ mice HSPC

Percentages of GFP+ (A) Gr-1^{+}, (B) Ter119^{+}, (C) CD41^{+}, (D) B220^{+}, (E) TCRβ^{+}, (F) LSK and (G) LK cells in the spleens of recipient mice are shown in bar graphs as mean ± SEM (n=6-8). (** p<0.01; *** p<0.001; **** p<0.0001). Statistical significances were determined using two-tailed unpaired t-test.

Supplementary Figure 5. S100A8 or S100A9 overexpression decreases myeloid and erythroid colony formation of Jak2$^{V617F/+}$ mice BM cells

(A, B) Hematopoietic progenitor colony formation assay. Lineage-negative cells from the BM of Jak2$^{V617F/+}$ mice were transduced with retroviruses expressing vector, S100A8 or S100A9. Infected cells were selected in puromycin for 48 hours, and 2.5×10^3 cells were plated in methylcellulose medium supplemented with cytokines. CFU-GM (A) and BFU-E (B) colonies were scored 7 days after plating. Data are shown in bar graphs as mean ± SEM (n=6 or 7). (**** p<0.0001). Statistical significances were determined using two-tailed unpaired t-test).

Supplementary Figure 1

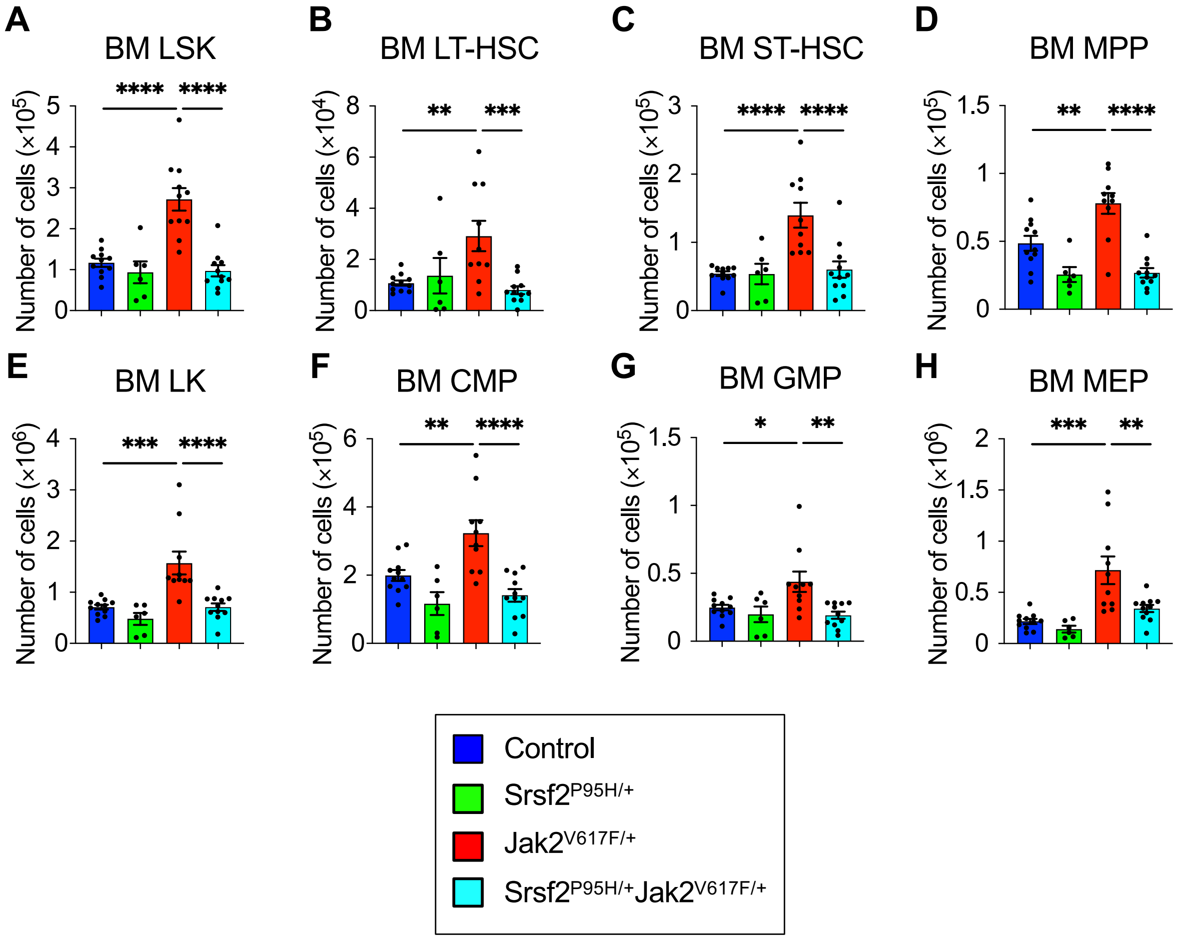

Supplementary Figure 2

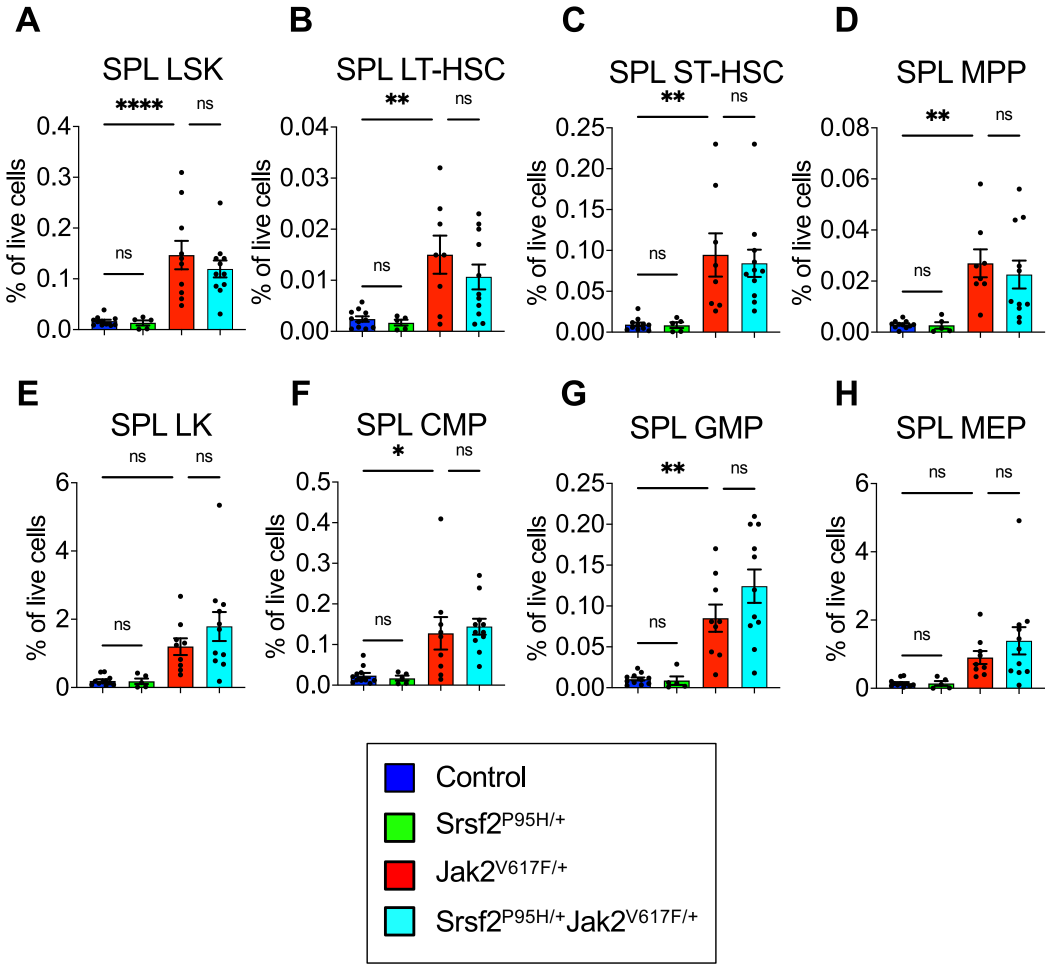

Supplementary Figure 3

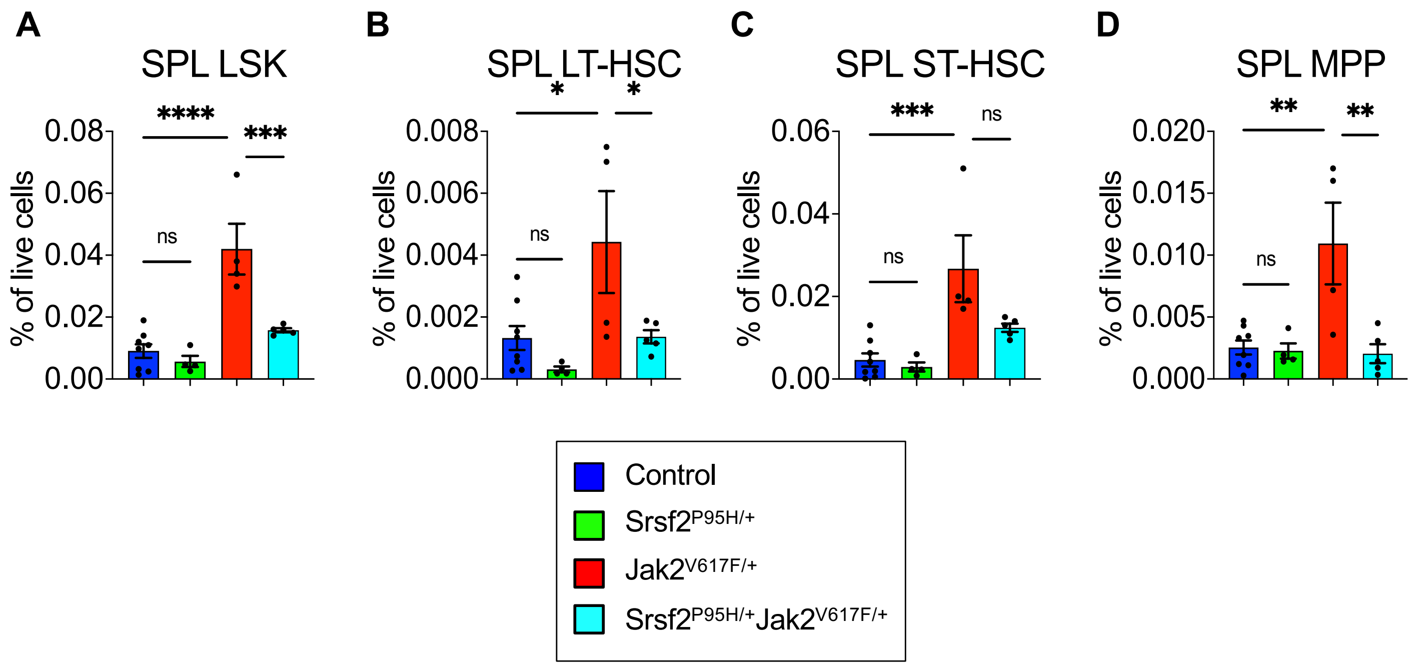

Supplementary Figure 4

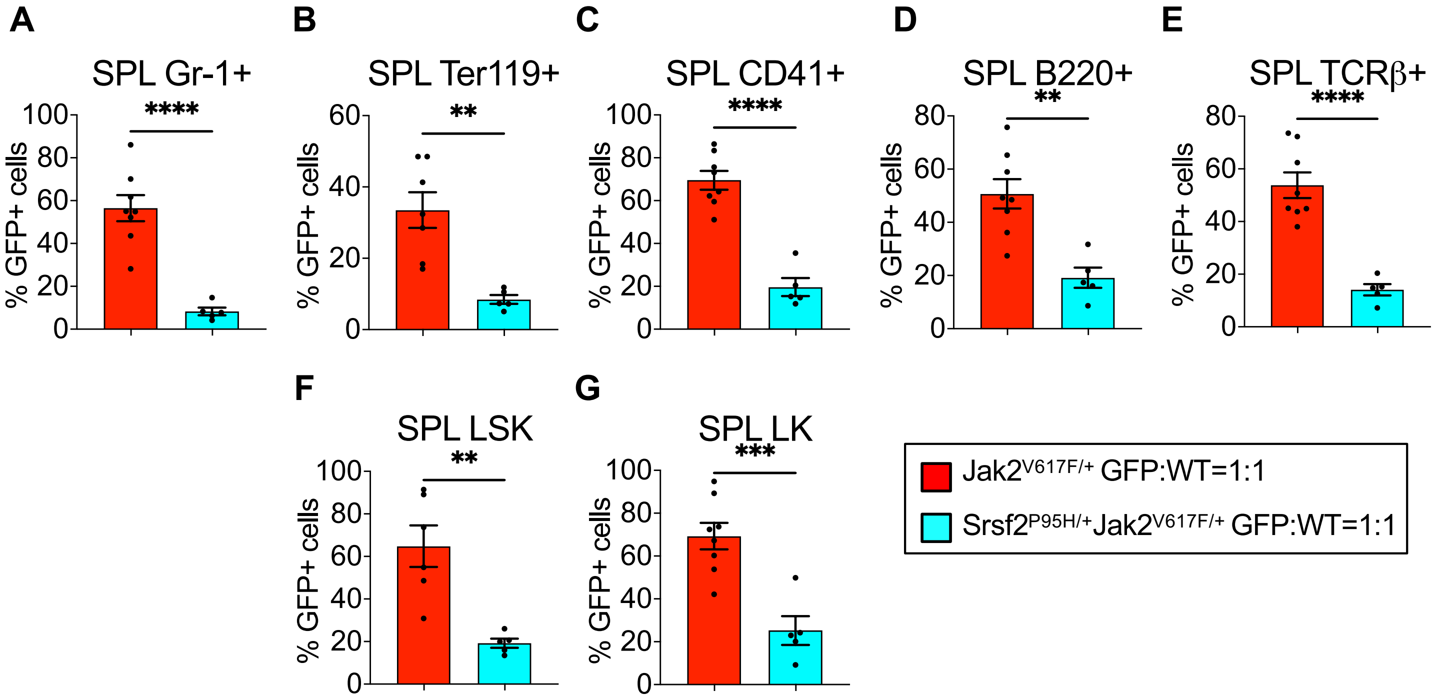

Supplementary Figure 5

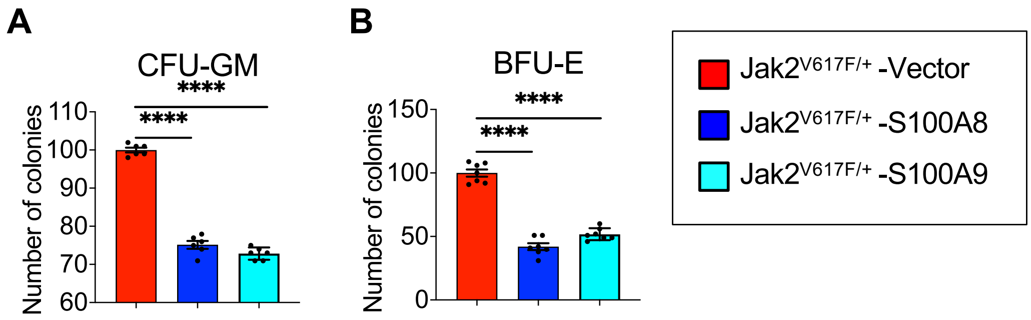

Supplement: Supplementary file 1 — Supplementary Information [file 41408_2023_947_MOESM1_ESM.pdf]
